# Cell-dependent antithrombotic effect of tranexamic acid

**DOI:** 10.3389/fimmu.2026.1820813

**Published:** 2026-05-26

**Authors:** Kata Balog Virág, Petra Csikós, Alexandra Raska, Barbara Baráth, Kristóf Molnár, Natalia Nikolova, Kiril Tenekedjiev, Krasimir Kolev, Nikolett Wohner

**Affiliations:** 1 Department of Biochemistry, Institute of Biochemistry and Molecular Biology, Semmelweis University, Budapest, Hungary; 2 HCEMM-SU Thrombosis and Hemostasis Research Group, Department of Biochemistry, Semmelweis University, Budapest, Hungary; 3 Department of Mechatronics, Faculty of Engineering, Nikola Vaptsarov Naval Academy, Varna, Bulgaria; 4 Department of Computer Science, Varna Free University “Chernorizets Hrabar”, Varna, Bulgaria; 5 Australian Maritime College, University of Tasmania, Launceston, TAS, Australia

**Keywords:** antithrombotic action, fibrinolysis, immunothrombosis, plasminogen, tranexamic acid

## Abstract

**Background:**

Tranexamic acid (TXA) is a synthetic lysine analog that inhibits fibrinolysis by blocking lysine-binding sites on plasminogen and plasmin. Although early therapeutic TXA reduces bleeding mortality in major trials, prophylactic benefit appears context- and timing-dependent. TXA is generally not associated with increased thromboembolism, yet its net effect on thrombus formation remains uncertain. Because plasmin(ogen) also acts on leukocytes, endothelium and platelets via cell-surface receptors, TXA may exert cell-dependent effects beyond antifibrinolysis.

**Objectives:**

To determine how cellular elements modulate TXA’s impact on thrombus formation.

**Methods:**

We studied TXA in an *in vivo* murine venous thrombosis model without endothelial injury (IVC stenosis). Plasma VWF: Ag and MCP-1 were measured by ELISA. Thrombin generation was assessed in whole blood and plasma-based systems to evaluate cell dependence. Leukocyte-associated plasminogen activation was quantified using plasmin generation assays in fibrin. Primary hemostasis was assessed by tail bleeding.

**Results:**

TXA reduced the odds of venous thrombus formation by 90% but did not alter thrombus mass once clots formed. VWF: Ag remained unchanged, whereas the stenosis-induced rise in MCP-1 was largely suppressed by TXA. TXA decreased thrombin generation in whole blood but not in platelet-rich plasma, indicating a cellular requirement. Consistently, TXA markedly inhibited leukocyte surface–mediated plasminogen activation within fibrin clots. Tail bleeding was unaffected.

**Conclusion:**

TXA was not prothrombotic in venous stasis; it reduced thrombus initiation without impairing primary hemostasis. We show for the first time that TXA modulates thrombin generation in a cellular environment, consistent with inhibition of leukocyte-associated plasmin activity.

## Introduction

Tranexamic acid (TXA) is a synthetic lysine analog that inhibits fibrinolysis by blocking the lysine-binding sites of plasminogen and plasmin. It is widely used to reduce bleeding in trauma, surgery and obstetrics. Large randomized trials in trauma (1) and postpartum hemorrhage (2) have shown that early therapeutic administration of TXA lowers death due to bleeding without increasing vascular occlusive events (3). At the same time, several recent studies have questioned how effective TXA really is when used prophylactically rather than as treatment. Prophylactic TXA did not reduce clinically relevant bleeding in thrombocytopenic patients with hematological malignancies in the A-TREAT and TREATT trials (4, 5), and large trials in vaginal birth and caesarean section have reported at most modest or no benefit of prophylactic TXA on postpartum hemorrhage (6). These findings suggest that the hemostatic effect of TXA is highly context- and timing-dependent: giving the drug shortly after bleeding starts appears beneficial, whereas routine prophylaxis before or in the absence of active bleeding often has little measurable effect.

Concerns about a possible prothrombotic effect of TXA have further complicated its use for prophylaxis. A large systematic review and meta-analysis of more than 200 trials concluded that intravenous TXA, across a wide range of indications and doses, is not associated with an increased risk of venous or arterial thromboembolism (7), in line with the neutral thrombotic signal in CRASH-2 and WOMAN trials (2, 3). In contrast, a nationwide Danish cohort study found a several-fold higher incidence of venous thromboembolism among women receiving oral TXA for heavy menstrual bleeding (8). Together, these data indicate that route of administration, dose, underlying disease and concomitant risk factors all influence how TXA affects thrombosis risk, and they underscore that its net prophylactic effect on thrombus formation remains uncertain.

Additional reassurance comes from large trials in high-risk cardiac surgery. In the ATACAS trial, more than 4,600 patients undergoing on- or off-pump coronary-artery bypass surgery were randomized to intravenous TXA or placebo; TXA significantly reduced bleeding and transfusion requirements, but the 30-day composite of death or thrombotic complications (myocardial infarction, stroke, pulmonary embolism, renal failure, or bowel infarction) did not differ between groups (9). Longer-term follow-up of the same cohort likewise showed no excess of death, severe disability, or major adverse cardiovascular events at 1 year in TXA-treated patients (10). Consistent findings have been reported in other CABG and off-pump CABG studies, in which perioperative TXA decreased postoperative blood loss and the need for allogeneic transfusion without increasing thromboembolic events or late cardiovascular outcomes (11–13). Although higher doses of TXA have been associated with an increased risk of postoperative seizures in cardiac surgery (9, 14), these regimens have not been accompanied by a clear increase in venous or arterial thrombosis.

Mechanistically, TXA is usually described as a purely antifibrinolytic drug, but plasmin(ogen) has important functions beyond fibrin degradation. Plasminogen receptors such as α-enolase, annexin A2/S100A10, histone H2B and Plg-RKT are widely expressed on monocytes, macrophages, neutrophils, endothelial cells and platelets, anchoring plasmin to cell surfaces and localizing its activity to pericellular microenvironments (15, 16). Many cell-surface plasminogen receptors engage plasminogen through lysine-dependent interactions and are therefore expected to be sensitive to inhibition by TXA. This applies to α-enolase, histone H2B and Plg-RKT, all of which expose or depend on C-terminal lysine residues, as well as to the annexin A2/S100A10 complex, in which the lysine-dependent plasminogen-binding function is attributable primarily to S100A10, not annexin A2 itself. Thus, TXA would be expected to inhibit plasminogen recruitment via α-enolase, S100A10, histone H2B and Plg-RKT. When bound to these receptors, plasmin acts as a potent cell activator: it induces chemotaxis and triggers intracellular signaling in monocytes and macrophages, including p38 MAPK and JAK/STAT pathways, leading to the production of pro-inflammatory mediators such as monocyte chemoattractant protein-1 (MCP-1), IL-6 and other cytokines (17, 18). MCP-1 in turn promotes recruitment and activation of inflammatory cells and has been linked to thrombo-inflammatory complications in several clinical contexts (19). These data support the concept of plasmin as a regulator of thrombo-inflammation, in which fibrinolytic and inflammatory pathways are tightly coupled through cellular elements.

Because TXA primarily inhibits fibrin-dependent plasmin generation by disrupting the lysine-dependent co-localization of plasminogen and tPA on the clot surface, it limits fibrinolysis at formed thrombi. However, by also preventing plasmin(ogen) binding to lysine-dependent cell-surface receptors, it is likely to interfere with cellular processes as well, although its effects beyond the clot surface may be more complex, including enhanced susceptibility of plasminogen to urokinase-type plasminogen activator (uPA)-mediated activation under some conditions (20, 21). Experimental work has shown that TXA can modulate complement activation and C5a generation in a plasmin-dependent, context-specific manner, and clinical as well as translational studies suggest that TXA has measurable immunomodulatory effects *in vivo (*22, 23). In patients undergoing major surgery, TXA has been reported to alter the immunophenotype of circulating phagocytes and to be associated with reduced postoperative infection rates, independent of its blood-sparing effect (24, 25). On the other hand, inhibition of plasmin by TXA did not measurably affect systemic inflammatory responses during experimental human endotoxemia (26), underscoring that the impact of TXA on cell-mediated inflammation is not uniform across immunological contexts. Overall, the available evidence indicates that the presence or absence of cellular elements and their interactions with plasmin are critical yet still poorly understood determinants of TXA’s net hemostatic and thrombotic effects.

In the present study, we investigate the effect of TXA on thrombus formation, with particular emphasis on how the presence or absence of cellular elements modulates its action.

## Methods

### Animals and anesthesia

Wild-type C57BL/6J mice (male, 8–12 weeks) were used for the infrarenal inferior vena cava (IVC) stenosis thrombosis model. Anesthesia was induced in an isoflurane chamber (3.5% isoflurane in O_2_, 1 L/min) and maintained via nose cone (2.5% isoflurane in O_2_, 1 L/min) for the duration of surgery. Core temperature was maintained on a heated pad, and ophthalmic ointment was applied to prevent corneal drying. Animals were housed under specific pathogen-free conditions with free access to food and water, and all procedures were conducted in accordance with institutional and national guidelines for animal care and experimentation, with prior approval from the local Scientific Ethical Committee on Animal Experimentation.

### IVC stenosis model

IVC stenosis was induced as previously described for a flow-restriction model of deep vein thrombosis in mice (27). In order to control for the known impact of venous side branches on thrombus formation in the IVC stenosis model, all visible side branches were ligated during surgery (28). At 48 h or 72 h post-surgery, the IVC segment between the renal veins and the iliac bifurcation was excised, and the formed thrombi were harvested for downstream analyses. The odds of thrombus formation (presence/absence) were calculated for all experimental groups.

### TXA administration

Mice received 10 mg/kg TXA intraperitoneally - in line with physiological, therapeutically relevant concentration used in humans - at 24 h and/or 48 h after surgery and 1 h before surgery. Control animals received vehicle (physiological saline) injections matched for volume and timing.

### Blood collection and plasma preparation

At the indicated time points (48–72 h post-surgery), immediately before thrombus retrieval, mice were anaesthetized with isoflurane and blood was collected from the retro-orbital venous plexus into 3.2% (w/v) sodium citrate (0.109 M) at a 9:1 (v/v) blood-to-anticoagulant ratio using glass capillaries, an approach recommended to minimize ex vivo platelet activation (29). Platelet-poor (PPP) and platelet-rich plasma (PRP) were prepared from citrated whole blood by sequential centrifugation. PRP was used immediately for the thrombin-generation assay, and PPP was stored at −80 °C until further analysis.

### VWF: Ag and MCP-1 quantification

High-binding polystyrene microplates were coated overnight at 4 °C with rabbit anti-VWF antibody (Dako, A0082; 1:500) in carbonate–bicarbonate buffer (10 mM NaHCO_3_/50 mM Na_2_CO_3_, pH 9.6). Plates were washed (PBS (8.1 mM Na_2_HPO_4_, 1.5 mM KH_2_PO_4_, 137 mM NaCl, 2.7 mM KCl, pH 7.4), 0.05% Tween-20) and blocked for 1 h at room temperature with 3% BSA in PBS containing 0.1% Tween-20. Plasma samples were diluted 1:10 in the blocking buffer and applied in triplicate. Bound antigen was detected with HRP-conjugated rabbit anti-VWF (Dako, P0226; 1:1000) prepared in PBS containing 0.1% BSA/0.1% Tween-20 for 1 h at room temperature. Color reaction was developed with 3,3′,5,5′-tetramethylbenzidine (TMB), the reaction was stopped with 0.18 M H_2_SO_4_, and absorbance was read at 450 nm (CLARIOstar, BMG Labtech, Ortenberg, Germany). VWF: Ag values were expressed relative to a pooled mouse plasma calibrator set at 100%.

Plasma MCP-1 concentrations were determined by sandwich ELISA using the Quantikine Mouse MCP-1 (CCL2/JE) kit (R&D Systems) according to the manufacturer’s instructions, with samples run in triplicate and concentrations calculated from a 4-parameter logistic standard curve.

### Thrombin generation assay

The endogenous thrombin potential (ETP) was determined by applying the approach originally developed for a chromogenic thrombin substrate in platelet-poor plasma (30) and later adapted for a fluorogenic substrate in platelet-rich plasma (31) and in whole blood (32) with some modifications. The estimation of the generated thrombin was based on the release of amino-4-methylcoumarin (AMC) from the fluorogenic thrombin substrate Z-Gly-Gly-Arg-AMC.HCl (GGR-AMC) (#4002155, Bachem AG, Bubendorf, Switzerland) continuously monitored by measuring the fluorescence (360 nm excitation and 450 nm emission) at 37 °C in a CLARIOstar Plus multi-mode plate reader, the high fluorescence gain of which allowed for reliable AMC detection at less than 1 µM concentration. The assay mixtures (100 µL volume) contained 360 µM GGR-AMC, whole blood (at 10% final hematocrit) or platelet-rich plasma (3×10^4^/µL final platelet count), rabbit brain thromboplastin (3,000-fold final dilution of Technoplastin, #5003009, Technoclone, Vienna, Austria), 11 mM CaCl_2_ in 10 mM HEPES 150 mM NaCl pH 7.4. The fluorescence signal showed linear dependence on AMC concentration in the range 1 – 36 µM, and in neither of our measurements did it exceed the signal for this upper limit (thereby, the consumption of GGR-AMC was less than 10% of its initial concentration in the assay mixture minimizing the potential internal filter effects). The concentration of free thrombin [Th] was calculated from a calibration curve of activity on GGR-AMC at 3 thrombin concentrations in the range 7–28 nM using human thrombin standard (01/578, National Institute for Biological Standards and Control, South Mimms, UK). The concentration of α_2_-macroglobulin-thrombin [a2MG-Th] was calculated from a calibration curve of activity on GGR-AMC at 3 concentrations in the range 7–28 nM using complexes of α_2_-Macroglobulin from human plasma (M6159, Merck Kft, Budapest, Hungary) with the thrombin standard pre-formed at a 2-fold molar excess of the inhibitor.

The first step in the analysis of the data was to convert the fluorescence signal to the AMC concentration [AMC] using the linear calibration described above. Thereafter, a piecewise smoothing algorithm was applied to remove noise arising from the aggregation of cellular elements in the assay mixture. For the smoothing, we built up two monotonically non-decreasing functions of [AMC] over time for each assay curve, as justified by the physical nature of the measurement – AMC is irreversibly formed and [AMC] cannot decrease. For the first monotonic function, each pair of subsequent points in the measured dataset was evaluated, the later point of each pair that generated a negative first derivative was excluded, the two closest measured points giving a non-negative derivative were connected by a straight line, and the points of this line corresponding to the time of the skipped points were imputed in the subset of function data. For the second monotonic function, each pair of subsequent points in the measured dataset was evaluated, the earlier point of each pair that generated a negative first derivative was excluded, the two closest measured points giving a non-negative derivative were connected by a straight line, and the points of this line corresponding to the time of the skipped points were imputed in the subset of the second function data. The two synthetic datasets generated in this way represented the maximum and minimum [AMC] values for each measured time point that satisfied the condition for a monotonically non-decreasing time course of [AMC] in each curve. Thus, it was justified to use the mean of the [AMC] values at each time point of the two monotonic functions to construct a smoothened, noise-free [AMC] progress curve, on which all subsequent analysis was performed to determine the Lag Time (LT), Time to Tail (TtT), Separation Constant (SC), and Endogenous Thrombin Potential (ETP). The LT was defined as the earliest time point of the smoothened [AMC] progress curve at which the rate of [AMC] increase exceeded the value of (10% of the maximal increase rate + 90% of the minimal increase rate). The TtT was defined as the earliest time point on the smoothed [AMC] progress curve after the point of maximal increase rate, at which the rate of [AMC] increase was less than the value (20% of the maximal increase rate + 80% of the minimal increase rate). We assumed that TtT marked the end of thrombin generation, and that after TtT, AMC was released only by the α_2_-macroglobulin-thrombin complex. The TtT value was used to split the smoothened [AMC] progress curve into two parts: [AMC] release by free thrombin, and [AMC] release by α_2_-macroglobulin-thrombin complex according to the algorithm described in Ref (33). For this splitting of the [AMC] progress curve into two components, the SC constant was determined from the slope of the [AMC] progress curve after TtT assuming that at any moment the [a2MG-Th] was proportional to the time integral of [Th], and the proportionality coefficient was this separation constant. Thereafter, the first derivative of the partial [AMC] curve attributed to free thrombin was converted to [Th] using the activity calibration with standard thrombin described above. The ETP was defined as the area under this thrombin generation curve between LT and TtT, as shown in the figures illustrating the results with this assay.

### Isolation of murine peripheral blood leukocytes

Peripheral murine blood was diluted 1:1 with PBS and carefully layered onto Ficoll-Paque PLUS (Cytiva) at a ratio of 4 parts diluted blood to 3 parts Ficoll. Samples were centrifuged for 35 min at 400*g* (maximum acceleration, minimum deceleration). The supernatant was gently aspirated until the red blood cell (RBC) fraction was reached, after which the uppermost layer of the RBC fraction was removed to minimize platelet contamination. The remaining RBC fraction was washed with Hanks’ Balanced Salt Solution without Ca^2+^ and Mg^2+^ (HBSS (136.9 mM NaCl, 5.4 mM KCl, 0.34 mM Na_2_HPO_4_, 0.44 mM KH_2_PO_4_, 4.17 mM NaHCO_3_, PH 7.2-7.4), Capricorn Scientific) by centrifugation for 10 min at 400*g*. Following centrifugation, the supernatant and the upper layer of the leukocyte-rich pellet were removed to eliminate residual platelets, and the pellet was resuspended in RBC lysis buffer (Roche). After 5 min incubation, cells were centrifuged for 5 min at 400*g*, and the supernatant was discarded. This lysis–centrifugation step was repeated at least twice until no visible RBCs remained. The final pellet was resuspended in 1 ml HBSS and centrifuged for 5 min at 400*g*. After removal of the supernatant, white blood cells (WBCs) were resuspended in 500 µl HBSS. WBC count was determined using an automated cell counter (Abacus Vet 5, Diatron) and confirmed by manual counting in a Bürker chamber.

### Plasminogen activation on the surface of peripheral leukocytes

Peripheral WBCs were incubated with human plasminogen (2 µM) in the presence or absence of 1 µM N-formyl-methionyl-leucyl-phenylalanine (fMLP) for 60 min at room temperature under gentle intermittent rotation. Cells were then washed and resuspended in HBSS containing 1.26 mM CaCl_2_, 0.81 mM MgSO_4_ and 0.49 mM MgCl_2_ (Capricorn Scientific). WBC suspensions were mixed with fibrinogen (2 mg/mL) and clotted by addition of thrombin (28 nM) in the presence or absence of TXA (4 µM) for 50 min at 37 °C. Control clots contained no WBCs or were not supplemented with plasminogen. Clot lysis was subsequently initiated by layering a solution containing 2 nM tissue-type plasminogen activator (tPA) and 0.5 mM fluorogenic plasmin substrate Boc-Glu-Lys-Lys-AMC (# 4007739, Bachem AG) on top of the clots. Plasminogen activation was quantified fluorometrically using a CLARIOstar spectrofluorometer (BMG Labtech) with excitation at 360 nm and emission at 450 nm in black, flat-bottom 96-well plates. The rate of plasminogen activation was quantified as the slope of the linear function relating fluorescence to time squared, as introduced previously for chromogenic assays (34). Independent experiments, each in 5 replicates, were analyzed. The relative efficiency of plasminogen activation was estimated from the ratio of the experimental slopes measured in the presence and absence of TXA.

### Tail bleeding assay

To assess the effect of TXA on primary hemostasis, mice were allocated to two main experimental conditions: operated to induce IVC stenosis and non-operated groups. Within each of these two conditions, animals were further subdivided into TXA-treated and untreated (vehicle) subgroups, yielding four experimental groups in total (non-operated + vehicle, non-operated + TXA, operated + vehicle, operated + TXA). The animals received systemic TXA or vehicle treatment as described in the TXA administration section above. The investigator performing the bleeding measurements was blinded to treatment allocation whenever possible.

Primary hemostasis was evaluated using a standardized tail bleeding assay adapted from Denis et al. and Lenting et al. (35, 36). Briefly, anesthesia for each experimental group was induced in an isoflurane chamber (3.5% isoflurane in O_2_, 1 L/min) and maintained via nose cone (2.5% isoflurane in O_2_, 1 L/min) for the duration of the tail bleeding assay. Subsequently, a controlled distal injury was created on the tail, and the bleeding tail was immediately immersed in pre-warmed physiological saline solution. Bleeding was monitored over 30 minutes. Cumulative blood loss was quantified during the observation window as a global readout of primary hemostasis.

### Statistical procedures

The effects of TXA on thrombus mass, VWF: Ag, and MCP-1 levels were analyzed with the Mann-Whitney U test for differences between TXA-treated and untreated groups, with cardinalities respectively *n*_1_ and *n*_2_. All the *n*_1_×*n*_2_ pairs of observations from the two samples were compared, and the test statistic *U* was estimated as the minimum of *U*_1_ and *U*_2_, where *U*_i_ is the count of cases in which an observation from group *i* is larger than the observation from the other group, plus half the count of the draws. The common language effect size *f*=*U*_1/(_*n*_1_×*n*_2)_ is an estimate of the probability that a random member from Population 1 will have a larger parameter than a random member from Population 2 (37). The statistical test was based on a Bootstrap procedure (with 10,000 pseudo-realities) that constructs the conditional distributions of *U* under the base (where the parameter distributions are the same) and the alternative hypotheses (where the parameter distributions differ). Applying a significance level of α=0.05, we estimated the probability of rejecting a true null hypothesis (p-value), the critical value (*U_crit_*), and the power (1−*β*) of the test. The bootstrap Mann-Whitney U test was performed using self-designed MATLAB R2024a (MathWorks, Natick, MA) software.

For statistical evaluation of differences in ETP across different regimens of TXA treatment groups of at least 3 and up to 10 mice were used, and the ETP was determined in at least 3 and up to 8 replicate measurements. To account for the different degrees of membership of the separate measured ETP values, these sets of observed ETP were treated as fuzzy samples, and four Bootstrap statistical procedures were implemented with 10,000 pseudo-realities (38) and equal sizes over empirical cumulative distribution functions as previously described (39). These procedures performed two-tailed and one-tailed tests for medians and a Kuiper test for distributions. All Bootstrap procedures calculated the probability of rejecting a true null hypothesis (p-value), and statistical significance for the difference in ETP between pairs of groups was identified if at least one test procedure for distribution and one test procedure for median of the synthetic datasets generated p-values lower than 0.05. For this statistical analysis, a self-designed software was implemented, in MATLAB R2024a (MathWorks, Natick, MA) using the MATLAB Statistics and Machine Learning Toolbox version 24.1.

For the statistical evaluation of the relative efficiency of plasminogen activation, the ratios of the experimental slopes measured in the presence and absence of TXA were treated as random variables, with the numerator and denominator measured with experimental error. A total of 10,000 Bootstrap pseudo-realities (pairs of synthetic samples of the ratios) were created by drawing with replacement from the experimental datasets (38). The P-values of 7 Bootstrap statistical tests were calculated: Kuiper Bootstrap test for distribution equality (39), two-tailed and one-tailed Bootstrap test for median equality (40), two-tailed and one-tailed Bootstrap test for bottom quartile equality, two-tailed and one-tailed Bootstrap test for top quartile equality (41). The test statistics for the respective Bootstrap hypothesis test were as follows: Kuiper’s statistics for the distribution test (42, 43), the difference of medians for the median test, and the difference of bottom and top quartiles for the bottom and top quartile tests, respectively. The applied approach for assessing the differences between two random variables based on two crisp samples addresses the debate around P-value validity (44). By using a cluster of tests instead of a single P-value, our conclusions gain additional statistical validity, and the likelihood of false-positive findings is substantially minimized, as previously used (40, 45, 46).

## Results

### TXA reduces the odds, but not the mass of venous thrombi in IVC stenosis model

**Figure 1:**
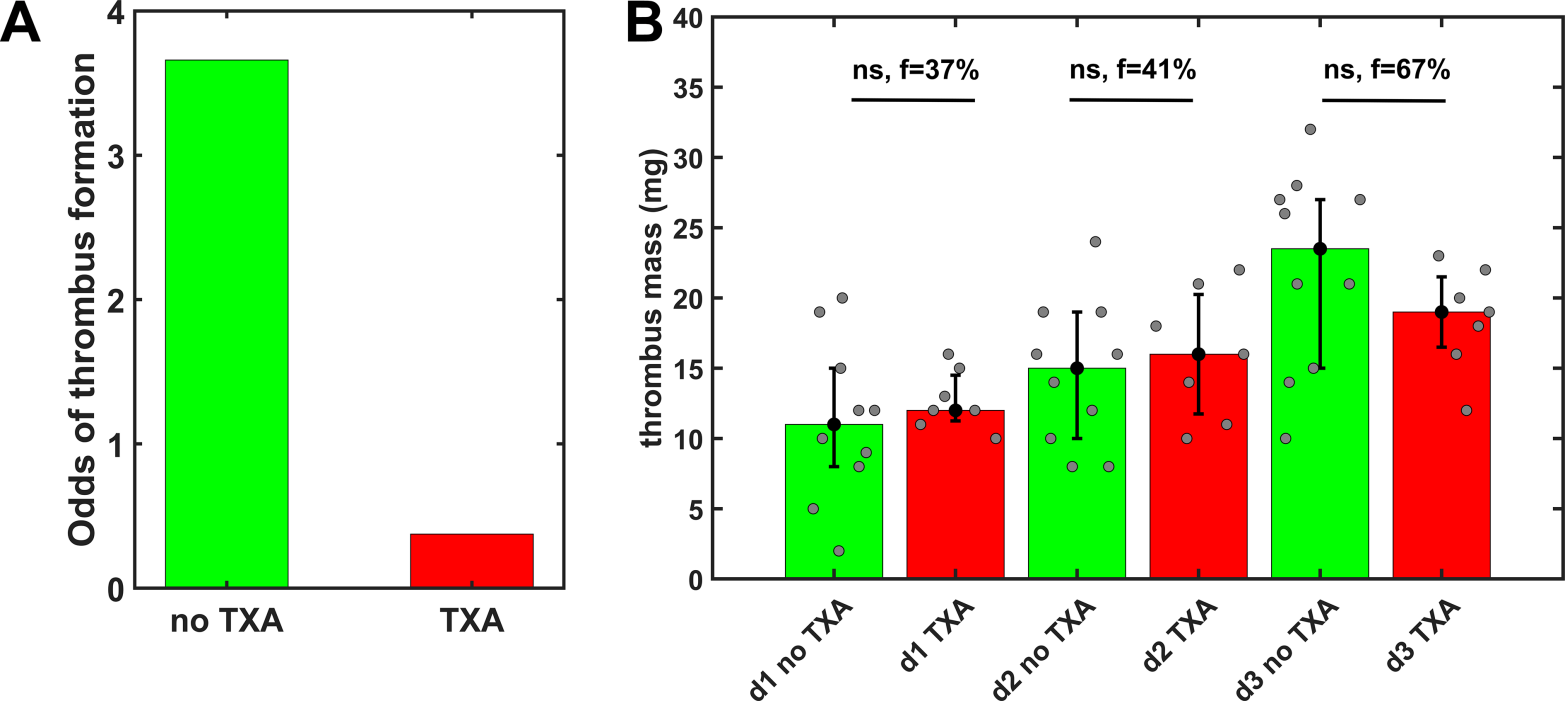
*In vivo* effect of TXA on thrombus formation following IVC stenosis. IVC stenosis was induced, and TXA was administered before and after surgery in mice as described in Materials and Methods. Each experimental group comprised at least 10 mice. **(A)** Odds of thrombus formation are shown as the number of thrombosed IVC stenosis events/the number of non-thrombosed IVC stenosis events observed totally on days 1 and 2 after surgery. **(B)** Mass of thrombi in IVC stenosis. If a thrombus was formed, its mass was measured 24 (d1), 48 (d2), or 72 (d3) hours after stenosis. The mass of at least 7 thrombi was analyzed per group. Bars indicate the median (bottom/top quartiles); symbols show individual data. Comparison of the indicated groups according to Mann-Whitney U test failed to reject the null hypothesis for the indicated pairs of groups (ns for p>0.05; f=common language effect size %).

In the IVC stenosis model, if TXA was administered before and after surgery as described in Methods, TXA markedly reduced the probability of thrombus formation (**Figure 1A**). The odds of thrombus formation were 3.66 in untreated mice and 0.375 in TXA-treated mice, corresponding to an odds ratio of 0.10 (Fisher’s exact test, p = 0.0172). Thus, prophylactic TXA was associated with an approximately 90% reduction in the odds of thrombus formation in the IVC stenosis model. However, if thrombi were formed, no significant difference was observed between the TXA-treated and untreated groups in the mass of thrombi removed 24–72 hours after stenosis, (**Figure 1B**).

### TXA does not alter the systemic VWF: Ag levels but suppresses the elevated MCP-1 levels in the IVC stenosis model

**Figure 2:**
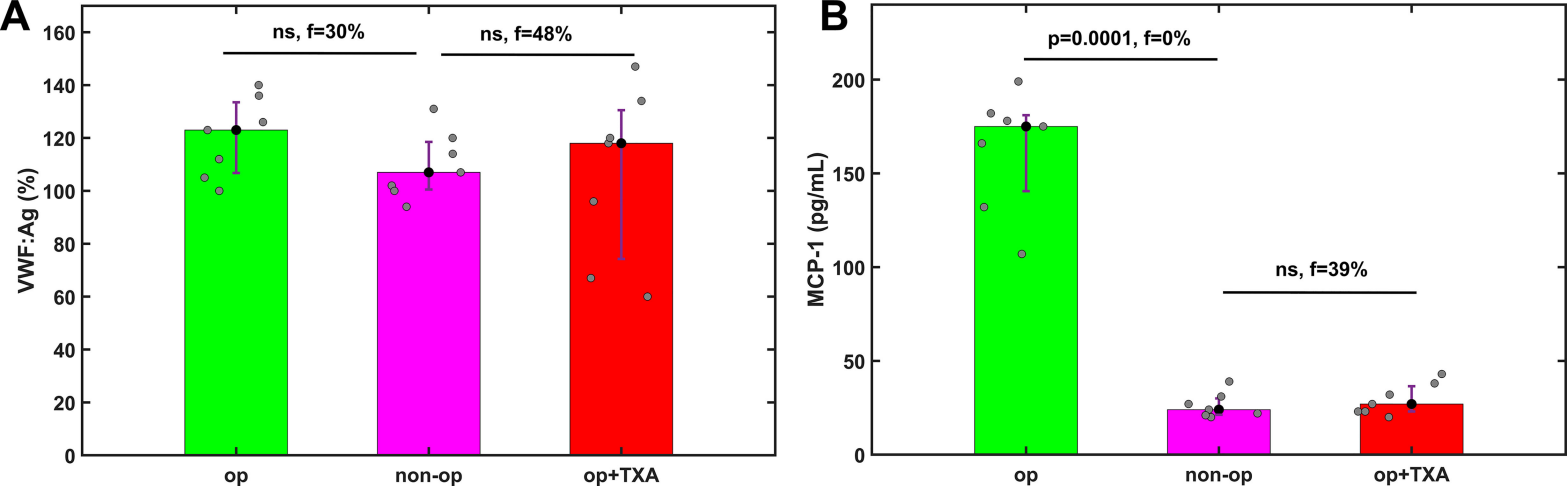
Effect of TXA on systemic markers of endothelial damage and inflammation in IVC thrombosis. Blood samples were taken at 48 hours after IVC stenosis (op) or from mice without surgery (non-op). TXA was administered 1 hour before and 24 hours after surgery (op+TXA). VWF: Ag **(A)** and MCP-1 **(B)** levels were measured in plasma samples as described in Materials and Methods. Median (bottom/top quartile) values are shown, symbols indicate individual data. P-value according to Mann-Whitney U test is indicated (ns for p>0.05; f, common language effect size %).

In line with the concept that the IVC stenosis model induces deep vein thrombosis primarily through flow restriction rather than overt endothelial denudation, plasma VWF: Ag levels were not significantly different between groups. In operated mice and TXA-treated operated mice VWF: Ag values remained in the range of non-operated controls (**Figure 2A**). By contrast, MCP-1 showed a clear inflammatory response to IVC stenosis, rising approximately six-fold in operated mice compared to the non-operated controls. TXA treatment largely abolished this increase, reducing MCP-1 concentrations in operated mice to values comparable to those seen in non-operated animals (**Figure 2B**).

### TXA reduces thrombin generation in whole blood, but has no effect in platelet-rich plasma

**Figure 3:**
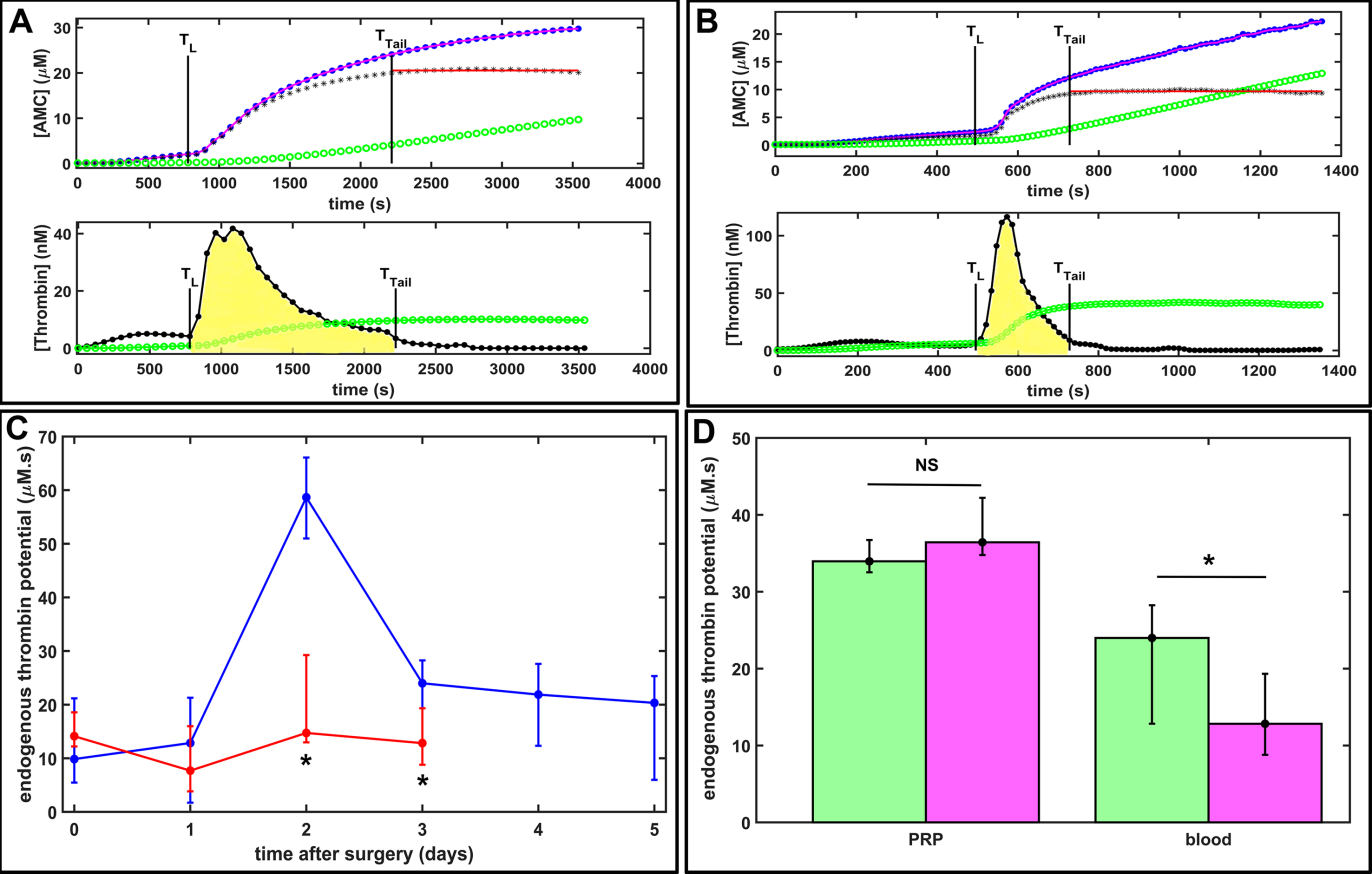
*In vivo* effects of TXA on the endogenous thrombin potential (ETP) of whole blood following induction of IVC thrombosis in mice. Standardized stenosis of IVC was used to induce thrombus formation in mice and blood samples were taken at daily intervals after the surgery for measurement of ETP. Blood samples from mice that were not exposed to surgery were used to determine the baseline parameters (on Day 0). All mice treated with TXA received a daily dose of 10 mg/kg body weight intraperitoneally (the first one immediately before the surgery). Thrombin generation was triggered by thromboplastin-CaCl_2_ in whole blood or platelet-rich plasma in the presence of Z-Gly-Gly-Arg-AMC thrombin substrate as described in Materials and methods. The fluorescence signal (360 nm excitation and 450 nm emission) of AMC released by thrombin was continuously measured and converted to AMC concentration. The blue symbols in the upper graphs of **(A, B)** illustrate the original traces of AMC release in whole blood 3 days after surgery in non-treated **(A)** and TXA-treated mice **(B)**. Applying the algorithms detailed in Materials and methods, these traces were smoothened (purple lines), the lag time (T_L_) and time-to-tail (T_Tail_) were determined, and the AMC curve was split in two parts for release by free thrombin (black symbols) and by thrombin in complex with α_2_-macroglobulin (green symbols). Thereafter, the first derivative of these two curves were calculated and converted to concentration of free thrombin and α_2_-macroglobulin-thrombin complex using a calibration [black and green lines, respectively, in the lower graphs of panels **(A, B)**]. The ETP was defined as the area under the thrombin curve between T_L_ and T_Tail_ shown in yellow. **(C)** Time course of ETP (median, interquartile range) in whole blood of mice after induction of IVC stenosis on Day 0 without TXA treatment (blue) or receiving once daily TXA (red). At each time point groups of 3 to 10 treated and untreated mice were compared using 3 to 8 replicate ETP measurements in each mouse. Bootstrap statistical procedures were implemented to analyze the differences in median and distribution between pairs of groups treated as fuzzy samples (asterisks indicate *p* < 0.05 for TXA-treated and untreated samples at the same time point). **(D)** ETP (median, interquartile range) in platelet-rich plasma (PRP) and whole blood of mice on Day 3 after induction of IVC stenosis without TXA treatment (green bars) or receiving once daily TXA (purple bars). Group sizes and statistical analysis were as in **(C)**.

The *in vivo* global potential to generate thrombin after induction of IVC stenosis was quantified by measuring the endogenous thrombin potential (ETP) in whole blood and PRP of mice with or without daily TXA treatment (10 mg/kg) (**Figure 3**). TXA administration moderated the ETP response following IVC stenosis. While surgery alone caused a 2- to 6-fold increase in whole-blood ETP on postoperative days 2 and 3, mice receiving TXA exhibited a suppression of the ETP curve relative to untreated animals. While TXA measurably reduced the thrombin-generating capacity of whole blood during the evolution of IVC thrombosis, it did not influence the postoperative ETP of platelet-rich plasma samples (**Figure 3D**). Therefore, the TXA effect on ETP could be attributed to blood cell-related, and not plasma- or platelet-dependent mechanisms.

### TXA inhibits plasminogen activation on the surface of isolated leukocytes in fibrin clots

**Figure 4:**
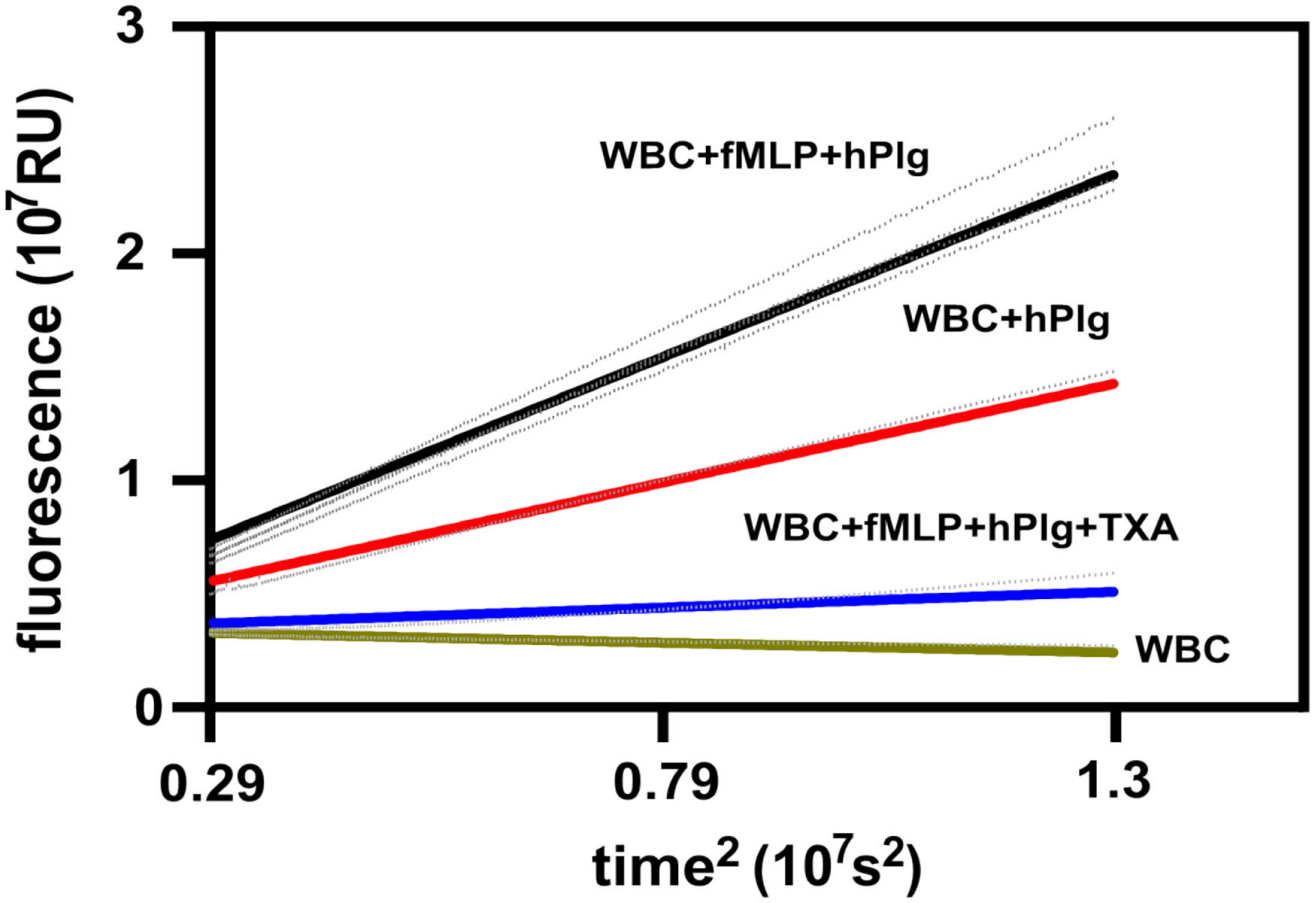
The effect of TXA on plasminogen activation on the surface of isolated leukocytes in fibrin clots. Fibrin clots containing isolated peripheral WBCs pre-coated with plasminogen (Plg) were prepared and plasminogen activation was initiated by tPA layered on the surface of the clots together with a fluorogenic plasmin substrate Boc-Glu-Lys-Lys-AMC. The fluorescence of the AMC product released by the generated plasmin was continuously measured and plotted versus time-squared yielding a linear relationship with a slope directly proportional to the rate of plasminogen activation. Solid lines represent linear regression to the mean of 4 replicates shown with dotted lines. WBCs were treated with fMLP at the time of plasminogen coating and TXA added to fibrinogen before clotting in the indicated samples.

Because of the cell-dependence of the TXA antithrombotic effects described above and the known inhibitory effect of TXA on plasminogen activation by tPA, it was of interest to evaluate the plasmin generation in the presence of WBCs. To this end, we modified our interfacial plasminogen activation assay (47) to incorporate isolated WBCs pre-coated with plasminogen as a single source of plasminogen in the fibrin clot (**Figure 4**). Without plasminogen supplementation, the isolated WBCs did not generate any plasmin activity in this assay. Stimulation of WBCs with fMLP strongly enhanced plasminogen activation. The ratio of plasmin generation rate with fMLP-treated cells to that with untreated cells had a median of 1.72 (IQR 1.61–1.93), presumably due to increased expression of plasminogen receptors and augmented binding of exogenous plasminogen. In the presence of TXA, cell-dependent plasminogen activation was markedly reduced. The ratio of activation velocities in the presence versus absence of TXA was used to quantify the strength of its modulatory effect. TXA reduced the activation rate with fMLP-treated WBCs to a 0.26 (IQR 0.22–0.29) median fraction of the rate with non-stimulated WBCs and a 0.15 (IQR 0.12–0.17) median fraction of the rate with fMLP-stimulated WBCs. Both the fMLP and TXA effects on plasminogen activation were statistically significant according to the cluster of hypothesis tests used instead of a single P-value, as detailed in Materials and Methods.

### IVC surgery and TXA treatment do not impair primary hemostasis

**Figure 5:**
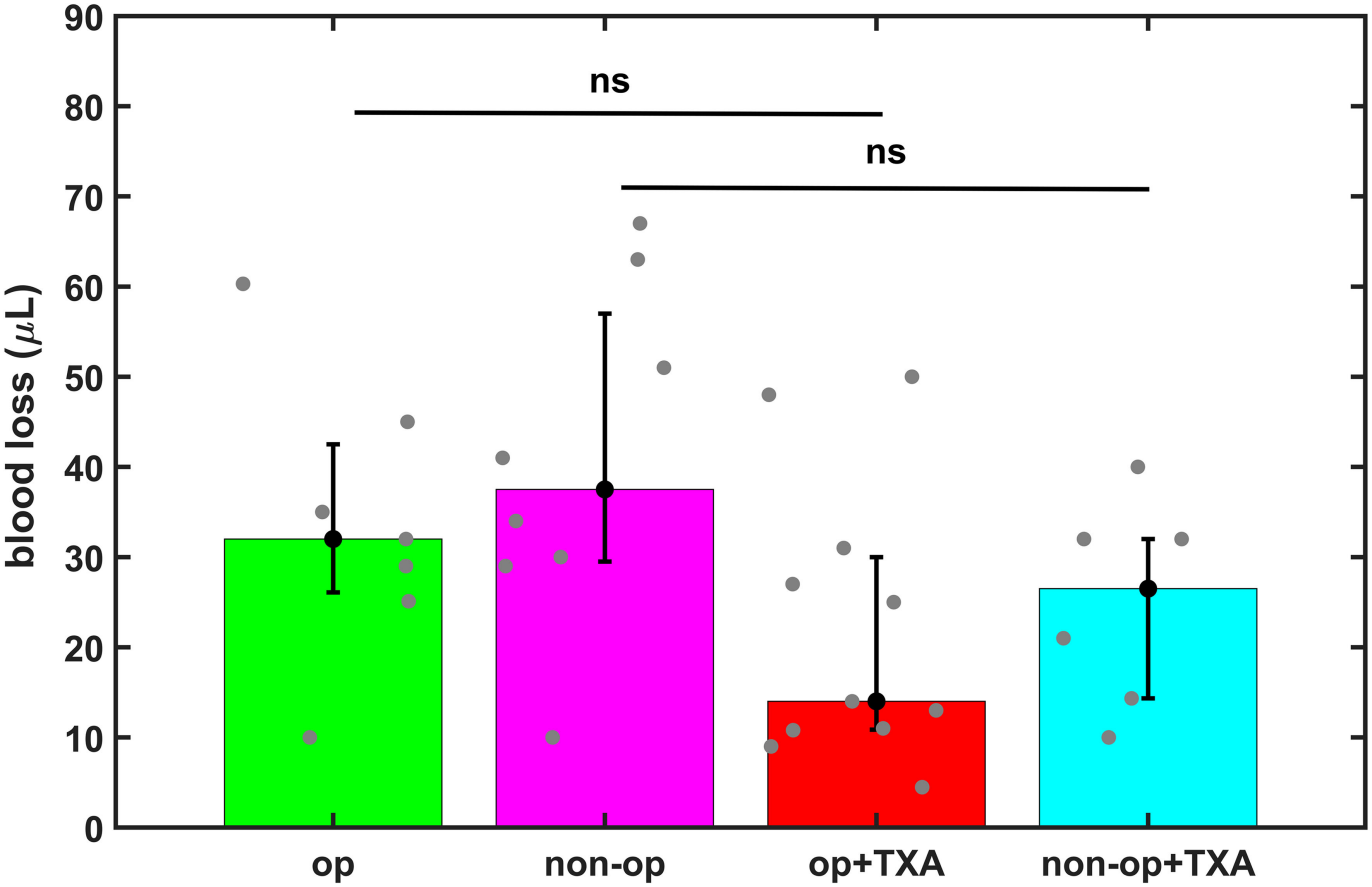
*In vivo* TXA effects on the global hemostatic capacity in mice. Mice were assigned to four groups: non-operated + vehicle (non-op), non-operated + TXA (non-op+TXA), IVC stenosis + vehicle (op), and IVC stenosis + TXA (op+TXA). IVC stenosis was induced and TXA-treated animals received systemic TXA before and after surgery, as described in Materials and Methods. Primary hemostasis was assessed by a standardized tail bleeding assay performed 48 hours after surgery, and cumulative blood loss over 30 minutes was recorded as a global measure of platelet- and VWF-dependent hemostatic function. Bars show the median (bottom/top quartile) values, symbols indicate individual data. No statistically significant differences in blood loss were observed between TXA-treated and untreated mice or between operated and non-operated groups (p>0.05 according to Kruskal-Wallis test for the indicated groups, ns).

Given the observed *in vivo* antithrombotic effects of TXA, its potential impact on the primary hemostatic mechanism was also plausible. Thereby, we evaluated the platelet- and von Willebrand factor–dependent hemostatic function by measuring the blood loss per unit time in a tail bleeding assay (**Figure 5**). In this experimental setting, no statistically significant differences in blood loss were observed between TXA-treated and untreated animals, nor between mice subjected to IVC stenosis and non-operated controls. These findings indicate that neither IVC surgery nor TXA administration measurably alters primary hemostasis.

## Discussion

TXA has traditionally been viewed as a purely antifibrinolytic drug that stabilizes fibrin clots and might therefore be expected to promote thrombosis. However, large randomized trials in trauma and postpartum hemorrhage have consistently shown that TXA reduces death due to bleeding without increasing major thromboembolic events, despite its potent inhibition of fibrinolysis. In the CRASH-2 trial (3) Early TXA administration in bleeding trauma patients significantly reduced all-cause mortality with no excess of vascular occlusive events. Similarly, in the WOMAN trial (2) TXA given within 3 hours after delivery decreased death due to postpartum hemorrhage without increasing thrombotic complications.

Additional clinical data support the notion that TXA is not prothrombotic even in patients at intrinsically increased thrombotic risk. In large arthroplasty cohorts, TXA has been shown to be safe in patients with pre-existing venous thromboembolism, coronary artery disease, coronary stents and, in many cases, prior coronary artery bypass grafting. For example, Zak et al. reported that intravenous or topical TXA in patients with coronary artery disease undergoing total joint arthroplasty did not increase the incidence of myocardial infarction, stroke or venous thromboembolism compared with controls (48). A recent systematic review and meta-analysis by Dang et al. likewise concluded that, in patients with pre-existing thromboembolic risk factors undergoing total joint arthroplasty, TXA reduced blood loss and transfusion requirements without increasing rates of deep-vein thrombosis, pulmonary embolism, or mortality (49). These findings are in line with earlier meta-analyses, such as that of Fillingham et al., which demonstrated that TXA use in hip and knee arthroplasty lowers transfusion rates without a detectable increase in thromboembolic events (50). Furthermore, large perioperative database studies examining myocardial infarction risk after TXA administration in surgical patients with prior coronary disease or coronary surgery have not identified an increased incidence of ischemic events attributable to TXA (51). Similarly, in a retrospective study of patients with malignancy undergoing orthopedic surgery, TXA was not associated with an increased risk of venous thromboembolism, despite the intrinsically high thrombotic risk of this population (52). Collectively, these data indicate that even in cohorts enriched for patients with previous coronary artery bypass graft and other cardiovascular comorbidities, perioperative TXA does not appear to precipitate additional thrombotic complications. In contrast, in the HALT-IT trial, a large randomized study in acute gastrointestinal bleeding, TXA did not reduce death due to bleeding and was associated with an increased risk of venous thromboembolic events. However, these findings should be interpreted in the context of a specific clinical setting - acute GI hemorrhage treated with a high-dose, 24-hour TXA infusion - which may limit generalization to other indications (53). These findings raise the possibility that the net *in vivo* effect of TXA on thrombosis and hemostasis is more complex than simply enhancing clot stability by inhibiting fibrinolysis.

In this context, we set out to investigate whether the antithrombotic profile suggested by clinical studies could be substantiated experimentally, with particular emphasis on cell-dependent mechanisms. Using the IVC stenosis model, we examined the *in vivo* effect of intraperitoneal TXA administered before and after surgery. Based on its well-known antifibrinolytic action, one might anticipate that TXA would increase the incidence or size of venous thrombi. Contrary to this expectation, TXA treatment in our model reduced the odds of thrombus formation, while the weight of thrombi that did develop did not differ between TXA-treated and untreated animals. Thus, in this murine venous thrombosis model, TXA behaved functionally as an antithrombotic agent with respect to thrombus initiation rather than a prothrombotic one.

Our findings are consistent with several mechanistic studies indicating that TXA does not augment thrombin generation when assessed in platelet-poor plasma. In the ETAPlaT substudy of the WOMAN trial, Dallaku et al. measured thrombin generation, platelet function and coagulation factors in women with postpartum hemorrhage randomized to TXA or placebo. They found no significant effect of TXA on endogenous thrombin potential or other thrombin generation parameters, nor on platelet function or coagulation factor levels, supporting the view that TXA has no major procoagulant effect in plasma (54). Likewise, Lisman et al. showed that although TXA dose-dependently inhibited fibrinolysis in pooled normal plasma, it did not change calibrated automated thrombography parameters (lag time, peak thrombin or ETP) regardless of whether coagulation was triggered via intrinsic or extrinsic pathways (55). Taken together, these studies argue against a plasma-driven prothrombotic action of TXA.

Our work extends this concept by demonstrating that the modulatory actions of TXA on coagulation become apparent in cell-rich systems. In whole blood from mice subjected to IVC stenosis, TXA reduced the endogenous thrombin potential, whereas thrombin generation in platelet-rich plasma was largely unaffected, indicating that the presence of blood cells is required for this effect. Furthermore, we observed that TXA significantly inhibited plasminogen activation on leukocyte surfaces, in line with recent data showing that TXA primarily targets cell-associated and surface-bound plasmin generation rather than bulk plasma fibrinolysis alone (25). Importantly, TXA did not impair primary hemostasis in the tail-bleeding assay, and IVC surgery itself did not alter tail bleeding. Thus, the antithrombotic effect we observed is unlikely to be mediated by modulation of the initial hemostatic response, but rather reflects downstream effects on coagulation, fibrinolysis and immunothrombosis, potentially through limiting cell-surface plasmin activity. Cell-associated plasmin has been shown to promote complement-dependent immunothrombosis, thereby exerting prothrombotic and pro-inflammatory effects *in vivo (*56). This interpretation is consistent with clinical data from the ETAPlaT substudy of the WOMAN trial, in which TXA had no measurable impact on platelet function assessed by multiple electrode aggregometry in women with postpartum hemorrhage.

These cell-directed effects of TXA are most likely mediated through inhibition of lysine-dependent plasmin(ogen) interactions at the cell surface, although not all plasminogen receptors are uniformly lysine-dependent, as discussed in detail in the Introduction. Moreover, additional off-target effects may arise at higher TXA concentrations, which were not examined in our experimental setting, as illustrated by the ATACAS trial, where higher TXA doses have been associated with inhibitory actions mediated through glycine and GABA receptors (9, 25).

Overall, our data support the notion that TXA exerts a cell-dependent antithrombotic effect under conditions in which leukocytes, platelets, and the vessel wall jointly regulate thrombin generation and fibrinolysis. Our study, to our knowledge, is the first to demonstrate that TXA alters thrombin generation in a cellular environment, showing a clear modulatory effect in whole blood that is not observed in platelet-poor plasma.

By attenuating cell-associated plasmin activity and modulating thrombin generation in whole blood without compromising primary hemostasis, TXA may shift the hemostatic balance toward effective control of bleeding while limiting pathological venous thrombosis. This cell-level perspective provides a mechanistic framework that helps reconcile the strong reduction in bleeding and the neutral thrombotic signal observed in large clinical trials with the absence of procoagulant effects in plasma-based assays, and suggests that TXA is not a universal prothrombotic agent but rather a context-dependent modulator of hemostasis.

In summary, our findings refine the current understanding of TXA by highlighting its predominantly cell-directed rather than plasma-driven actions on the coagulation–fibrinolysis axis. By demonstrating that TXA limits leukocyte-associated plasmin activity and modulates thrombin generation in whole blood without impairing primary hemostasis, this work provides a mechanistic link between experimental observations and the neutral or even protective thrombotic profile of TXA reported in large clinical trials. These results argue against viewing TXA as a broadly prothrombotic agent and instead support a context-dependent role in stabilizing hemostasis while restraining immuno-thrombosis in settings of venous stasis and inflammation. Future studies should clarify how these cell-level effects translate across different thrombotic phenotypes and dosing regimens, and whether they can be therapeutically exploited beyond classical bleeding indications.

## Data Availability

All raw data analysed in this publication are deposited in the Zenodo public repository under DOI 10.5281/zenodo.20187857: https://zenodo.org/records/20187857.
