# Supplementary material for: Cell-dependent antithrombotic effect of tranexamic acid
Source: Front Immunol. 2026 May 26;17:1820813. doi: 10.3389/fimmu.2026.1820813 (PMC13246336; doi:10.3389/fimmu.2026.1820813)
Supplement: Supplementary file 1 [file DataSheet1.docx]

**Supplementary data**

**Cell-dependent antithrombotic effect of tranexamic acid**

**Kata Balog Virag, Petra Csikós, Alexandra Raska, Barbara Baráth, Kristóf Molnár, Natalia Nikolova, Kiril** Tenekedjiev**, Krasimir Kolev, Nikolett Wohner**

Mann-Whitney U test for differences between the compared groups

The test statistic *U* was calculated using a Bootstrap procedure (with 10,000 pseudo-realities) that constructs the conditional distributions of *U* under the null hypothesis (identical parameter distributions) and the alternative hypothesis (different parameter distributions), as described in the Methods section. The figures show the cumulative density functions (CDFs) of the two *U* statistics, the estimated probability of rejecting a true null hypothesis (*p*-value), the common language effect size (*f*), the observed *U* value (*U_obs_*), the critical *U* value (*U_crt_*), and the power (1−*β*) of the test applying a significance level of *α*=0.05 for the indicated pairs of groups. The low power of the test – except in Figure S6 – is based on the similarity of the two CDFs, which have a very small median shift. In such cases, it cannot be substantially increased with a reasonable increase in the sample size. Although the Mann-Whitney U test can be used with as few as 5 observations in one of the samples, it is generally recommended that at least 7 observations be used to obtain robust, appropriate test results.[Nachar, N. (2008)].

Nachar, N. (2008). The Mann-Whitney U: A test for assessing whether two independent samples come from the same distribution. *Tutorials in quantitative Methods for Psychology*, *4*(1), 13-20. DOI:10.20982/tqmp.04.1.p013


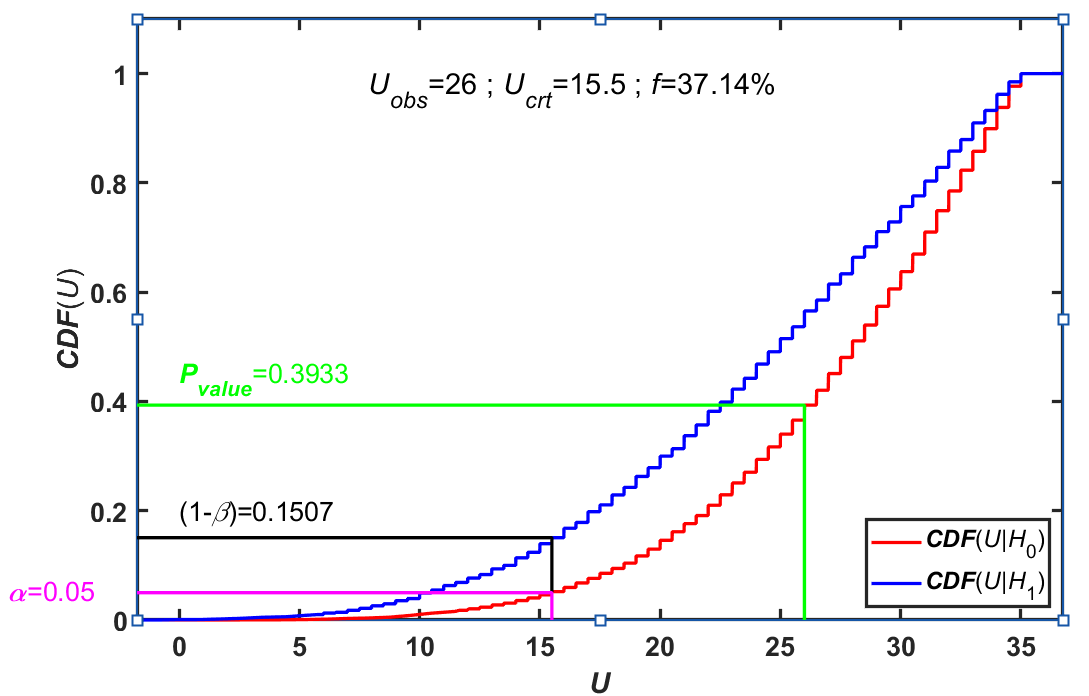


Figure S1. Comparison of thrombus mass in TXA-treated and untreated groups 1 day after surgery.


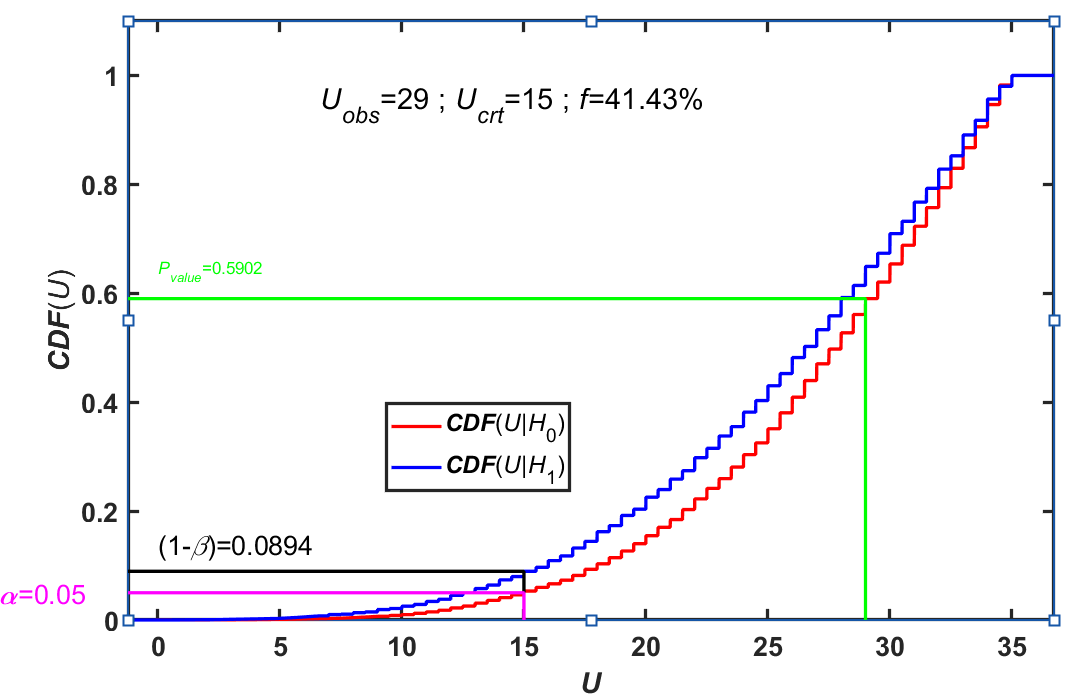


Figure S2. Comparison of thrombus mass in TXA-treated and untreated groups 2 days after surgery.


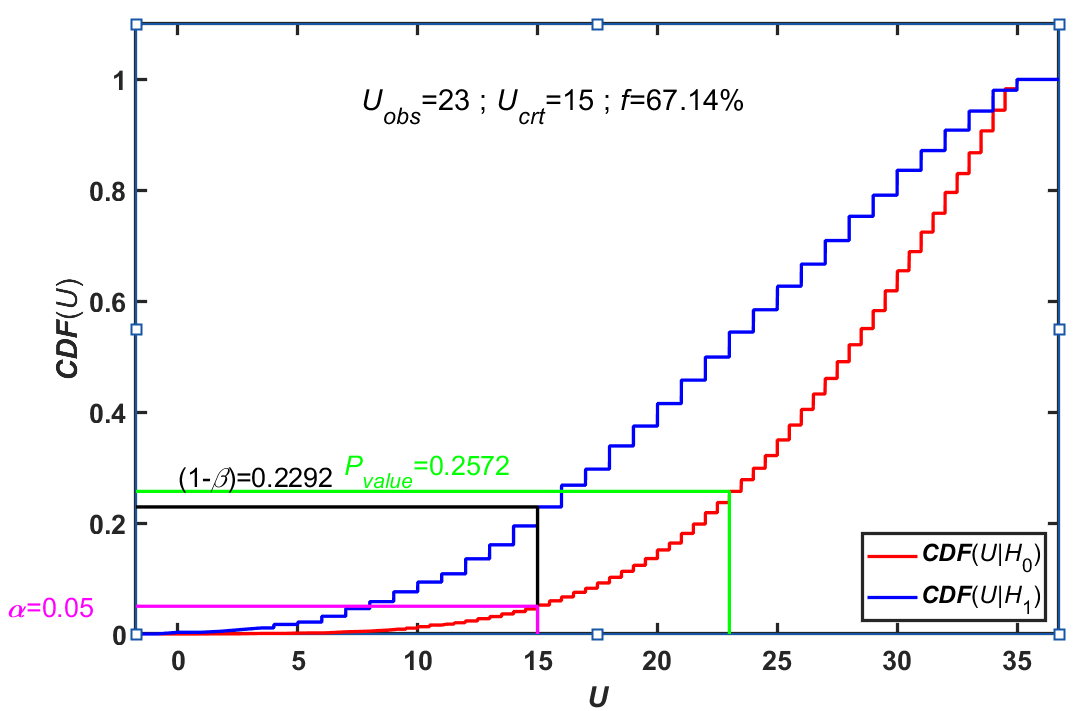


Figure S3. Comparison of thrombus mass in TXA-treated and untreated groups 3 days after surgery.


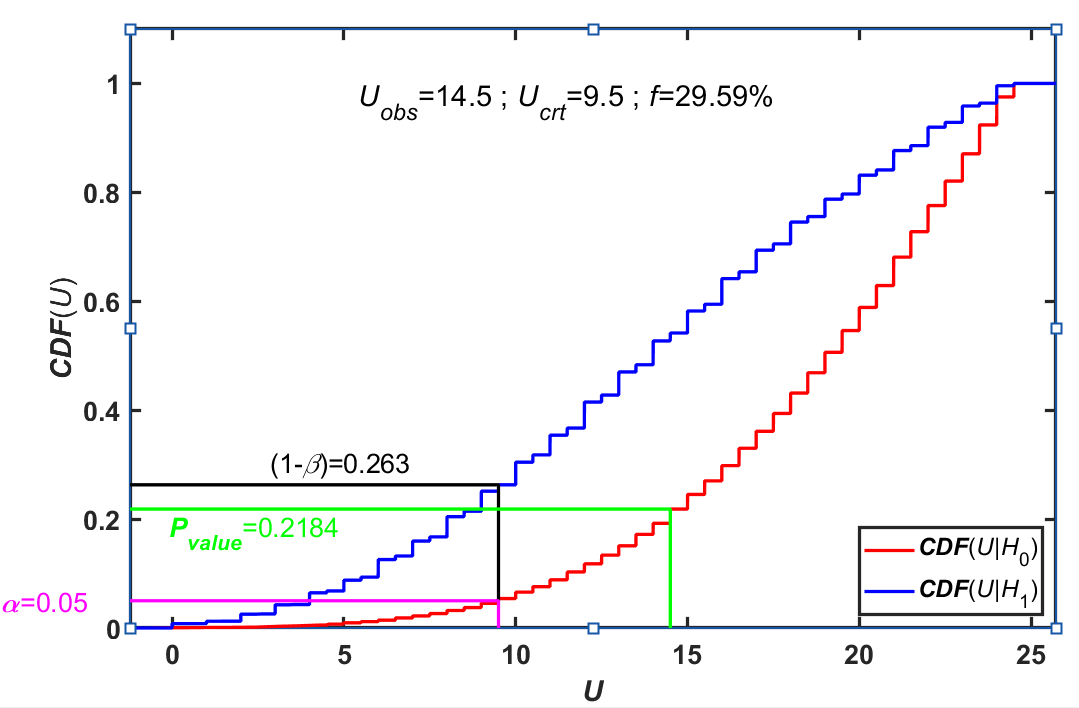


Figure S4. Comparison of VWF antigen levels before and after surgery.


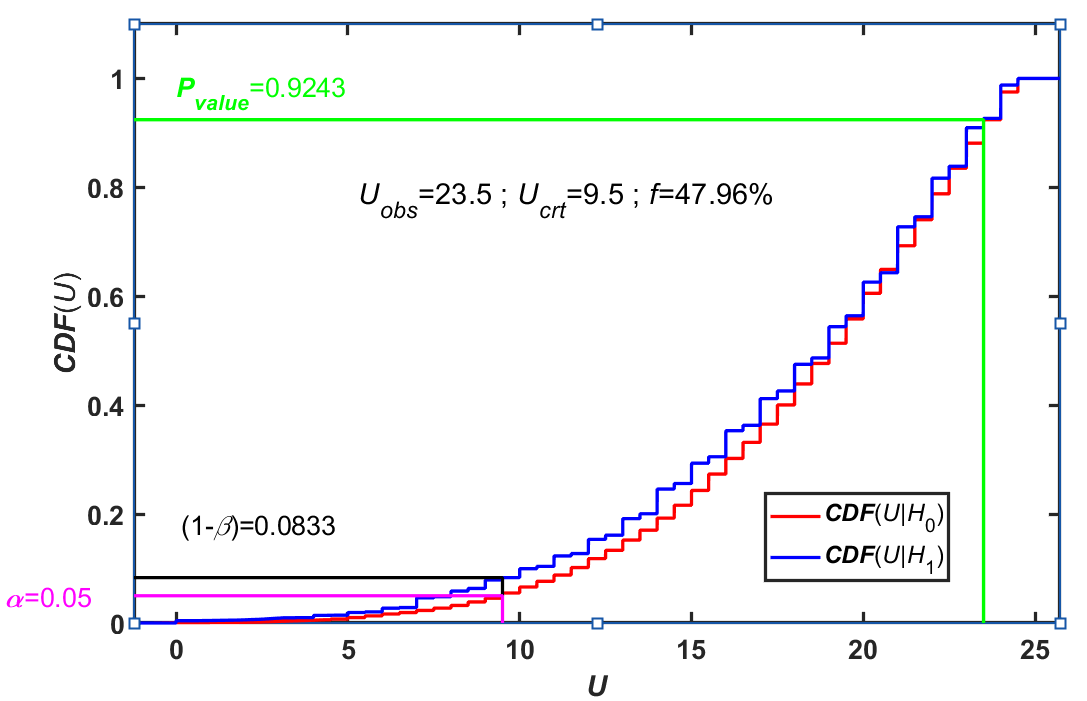


Figure S5. Comparison of VWF antigen levels before surgery and following tranexamic acid treatment after surgery.


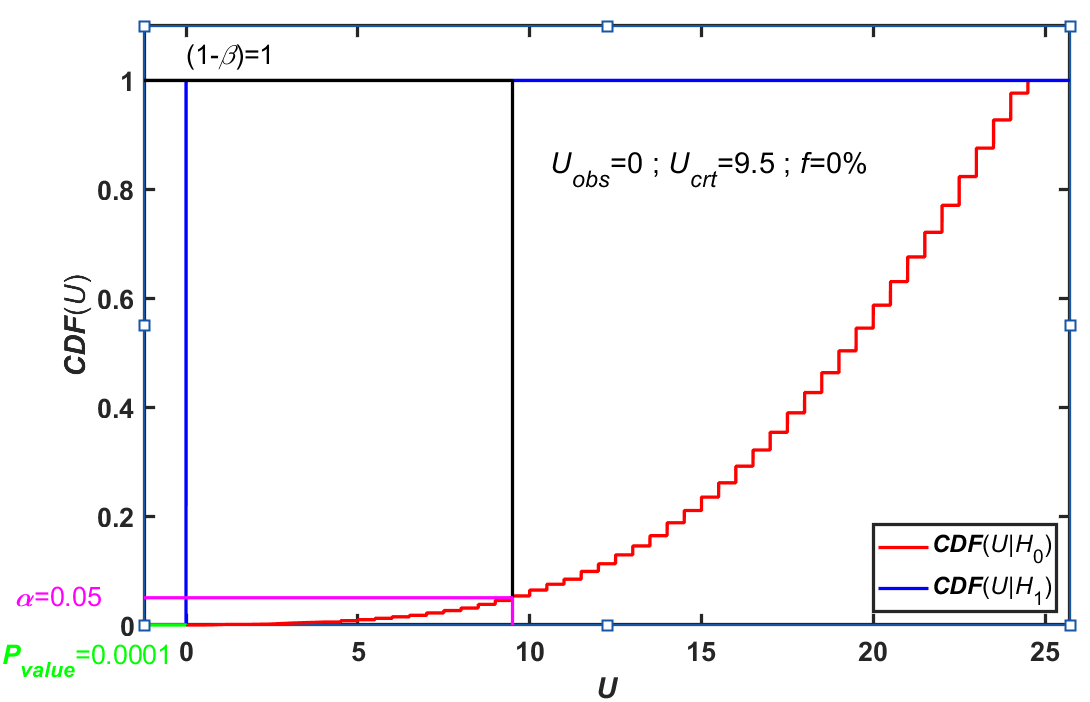


Figure S6. Comparison of MCP-1 levels before and after surgery.


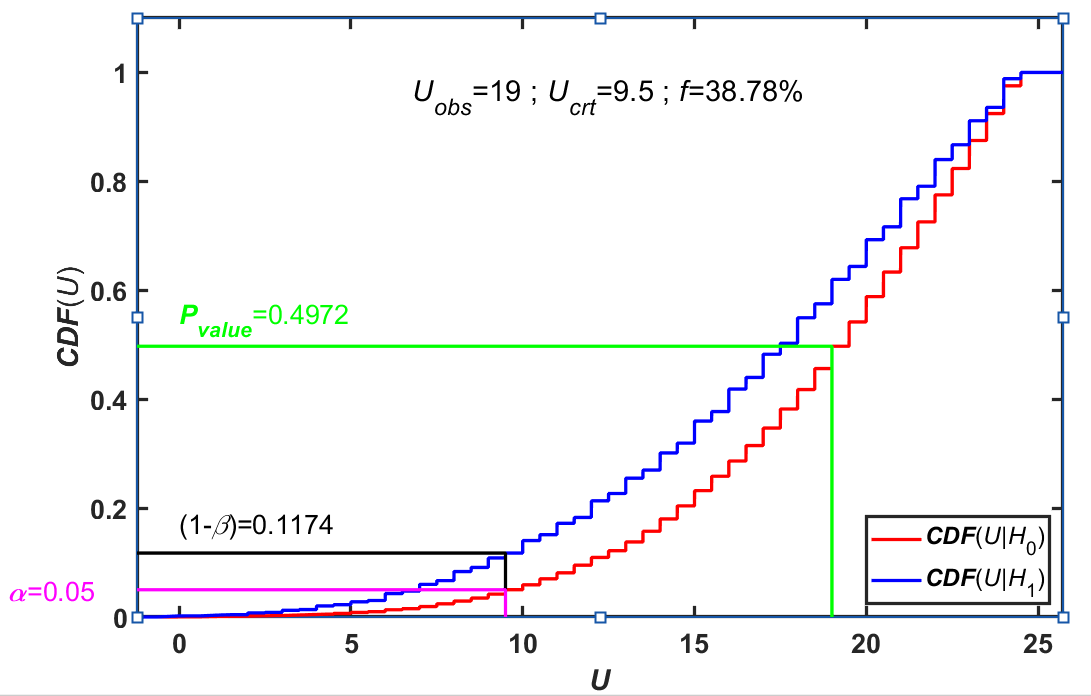


Figure S7. Comparison of MCP-1 levels before surgery and following tranexamic acid treatment after surgery.
